# What stops children with a chronic illness accessing health care: a mixed methods study in children with Chronic Fatigue Syndrome/Myalgic Encephalomyelitis (CFS/ME)

**DOI:** 10.1186/1472-6963-11-308

**Published:** 2011-11-11

**Authors:** Carly M Webb, Simon M Collin, Toity Deave, Andrew Haig-Ferguson, Amy Spatz, Esther Crawley

**Affiliations:** 1 St George's University of London, Cranmer Terrace, London, UK; 2 Centre for Child and Adolescent Health, School of Social & Community Medicine, University of Bristol, Bristol, UK; 3 Centre for Child and Adolescent Health, University of the West of England, Bristol, UK

## Abstract

**Background:**

Paediatric Chronic Fatigue Syndrome/Myalgic Encephalomyelitis (CFS/ME) is relatively common and disabling with a mean time out of school of more than one academic year. NICE guidelines recommend referral to specialist services immediately if severely affected, within 3 months if moderately affected and within 6 months if mildly affected. However, the median time-to-assessment by a specialist service in the UK is 18 months. This study used a mixed-methods approach to examine factors associated with time taken to access specialist services.

**Methods:**

Time-to-assessment was analysed as a continuous "survival-time" variable in Cox regression models using data from self-completed assessment forms for children attending a regional specialist CFS/ME service between January 2006 and December 2009. Semi-structured interviews about barriers experienced in accessing healthcare for their child were conducted with nine parents of children aged < 17 years (8 individual and one parent couple). Interviews were digitally recorded and analysed using "thematic analysis".

**Results:**

405 children were assessed between 2006 and 2009 and information on school attendance was available on 388. Only 1/125 with severe CFS/ME and 49/263 (19%) with mild to moderate CFS/ME were seen within NICE recommended timeframe. Increased fatigue was associated with shorter time to assessment (HR = 1.15; 95% CI 1.03, 1.29 per unit increase in Chalder fatigue score; P = 0.01). Time-to-assessment was not associated with disability, mood, age or gender. Parents described difficulties accessing specialist services because of their own as well as their GP's and Paediatrician's lack of knowledge. They experienced negative attitudes and beliefs towards the child's condition when they consulted GPs, Paediatricians and Child Psychiatrists. Parents struggled to communicate an invisible illness that their child and not themselves were experiencing.

**Conclusions:**

GPs, Child Psychiatrists and Paediatricians need more knowledge about CFS/ME and the appropriate referral pathways to ensure timeliness in referral to specialist services.

## Background

Paediatric chronic fatigue syndrome, or myalgic encephalomyelitis/encephalopathy, (CFS/ME), is defined as "generalised fatigue persisting after routine tests and investigations have failed to identify an obvious underlying cause" [1]. It is relatively common in young people (prevalence 0.4% to 2.0%) [2-4] and is very disabling, with a mean time out of school estimated at more than one academic year [5]. Over half the children accessing a specialist paediatric CFS/ME service in the UK attended one day or less of school each week [6].

In 2007, the National Institute of health and Clinical Excellence (NICE) recommended that children who were severely affected (housebound) with CFS/ME should be offered referral to specialist services immediately, those that were moderately affected (missing significant amounts of school) should be referred after 3 months of symptoms and those that had mild CFS/ME (attending full time school) should be offered referral after six months of symptoms [7]. This advice is consistent with the Chief Medical Officer's report [8], Royal College of Paediatrics and Child Health (RCPCH) guidelines [1] and the Department of Health exemplar for the management of CFS/ME [9]. Despite this, access to specialist help can take a considerable length of time, with a median time to assessment by a specialist service in the UK of 18 months [6].

Little is known about why parents experience delays in accessing specialist care for their children with CFS/ME. This study used a mixed-methods approach to examine the factors associated with time taken to access specialist services and explore the issues experienced by parents prior to assessment in a specialist service.

## Methods

### Patient cohort

The Bath specialist paediatric CFS/ME service, based at the Royal National Hospital for Rheumatic Diseases (RNHRD), provides a local service for a region in the South West of England which includes both rural and urban populations. Specialist clinics are located in Bath, Bristol, Gloucester, Bridgwater, and Swindon. Children are offered domiciliary assessment if they are unable to travel to a clinic. The region has a population of some 400,000 children aged between 5 to 19 years (2001 census). Our study was based on children ≤ 18 years old with a diagnosis of CFS/ME confirmed at assessment during the period between 1^st ^January, 2006 and 31^st ^December, 2009.

### Inventories

All children seen by the specialist CFS/ME service completed five inventories prior to assessment. Fatigue was measured using the 11-item Chalder Fatigue Questionnaire [10], scored using the 0-3 method (0 for "Less than usual", 1 "No more than usual", 2 for "More than usual" and 3 for "Much more than usual"). Functional disability was measured using the 10-item SF-36 physical function subscale [11]. In the SF-36, children's responses were scored between 1 ("Yes, limited a lot") and 3 ("No, not limited at all") for each question, so that children with the worst physical function scored 10 while those with good physical function scored 30. Two inventories were used to screen for mood problems, the Hospital Anxiety and Depression Scale (HADS) [12] and the Spence Children's Anxiety Scale (SCAS) [13]. The HADS is a 14-item questionnaire (comprising 7-item anxiety and depression subscales) given only to children >12 years old). The SCAS is a 38-item scale (with an additional 6 filler questions) which measures the frequency with which a child experiences symptoms relating to anxiety. Each question is scored as "Never" (0), "Sometimes" (1), "Often" (2) and "Always" (3). Pain was measured using a Visual Analogue Pain Rating Scale with a score of 0 for "no pain" and 100 for "pain as bad as possible". Inventories were coded as missing if >1 question was missing, apart from the SCAS, which was coded as missing when there were >2 missing items. On the HADS, each 7 item subscale was excluded if there was more than 1 question missing. Questions where two responses were given were coded as missing. Total scores were corrected for the number of missing items.

At assessment, children were asked about symptoms associated with CFS/ME [14]. Time-to-assessment was obtained from a question asking how many months had elapsed since the onset of symptoms. School attendance was recorded using a single question asking "How would you describe your attendance at school or college?" with responses of "None", "About 10% (e.g. one half day)", "About 20% (e.g. one day)", "About 40% (e.g. two days)", "About 60% (e.g. three days)", "About 80% (e.g. four days)", "Full time (100%)", and "Not applicable". In accordance with NICE guidelines, children were defined as severely-affected if they were unable to attend school; moderately-affected if school attendance was between 20-60% and mildly-affected if school attendance was 80-100% [7].

### Statistical analysis

We used Student's t test to compare mean values of continuous measures (age, fatigue, SF-36, total number of symptoms, depression, and anxiety) between the two groups of children whose mothers were interviewed and whose mothers were not interviewed. We used Fisher's exact test to compare proportions between these two groups for sex and severity of illness. We used the Mann-Whitney two-sample test to compare time-to-assessment. We used time-to-assessment as a continuous "survival-time" variable in a multivariable Cox regression model to identify patient characteristics that were independently associated with time-to-assessment. So that hazard ratios for the different inventories were comparable, inventory scores were rescaled so that the range for each was approximately 0-10. Thus, HADS anxiety and depression were divided by 2; SCAS and pain were divided by 10; and SF-36 and Chalder Fatigue were divided by 3. All analyses were restricted to children with no missing data in any of the variables investigated. The data were analysed using STATA 11 (StataCorp, College Station, TX, USA).

### Qualitative methods

Parents of children with CFS/ME under 16 years of age with a confirmed diagnosis of CFS/ME were recruited consecutively when attending either assessment or follow-up at the RNHRD by the specialist CFS/ME clinician between November and December 2010. Parents were invited to participate by a member of the clinical team at the end of the child's appointment and were given an information sheet and consent form at the end of the consultation. Each parent who had consented or who had agreed verbally to take part was telephoned over 24 hours later by CW to arrange an interview at a time and place that was convenient for the parents. Interviews were all conducted in the RNHRD, as requested by parents and lasted between 30 - 40 minutes.

### Semi structured interview

The content of the semi-structured interviews was initially based on a review of literature and then amended with advice from the Association for Young People with ME (AYME). The purpose of the interviews was to explore the barriers to accessing healthcare experienced by parents of children with CFS/ME. Interviews were digitally recorded and transcribed verbatim by CW. Individual numerical codes were used to protect confidentiality and names were changed within the text.

### Qualitative analysis

Each interview tape was listened to and transcripts read several times to develop a sense of the content. The data were analysed manually using content analysis after categorisation into main sub-headings [15,16]. A thematic analysis was then conducted [15]. Specifically, themes were identified in a semi-deductive manner where codes were identified from adult CFS/ME literature and compared with themes that emerged from our data. This included salient ideas, concerns and perceptions from different interviews being grouped together to form meaningful themes. Ideas that emerged from our data were grouped into a thematic framework including Global Themes, Sub-Themes, Codes and Sub-codes. Themes were identified and compared by two independent researchers (CW, AH-F). The interviews were re-visited by TD for final coding.

### Rigour

Data validation was achieved by feeding the themes back to AYME. They confirmed our findings and reiterated that health professionals need more knowledge about CFS/ME information. The findings were also fed back to the clinicians to ascertain whether they considered that the themes reflected the reality of these parents' experiences. The clinicians considered that the aspects highlighted by the parents were probably an accurate reflection of parents' experiences elsewhere in the UK.

### Ethical approval

Ethical permission for the qualitative part of the study was granted by the North Somerset and South Bristol NHS Research Ethics committee (REC Reference number 09/H0106/81). The study was also approved by the Research and Development department of the RNHRD. The North Somerset & South Bristol Research Ethics Committee decided that the collection and analysis of data collected routinely on all children was part of service evaluation and as such did not require ethical review by a NHS Research Ethics committee or approval from the NHS R&D office (REC reference number 07/Q2006/48).

## Results

405 children (aged 2 to 18 years) were assessed by the Specialist Paediatric CFS/ME service between 01/01/2006 and 31/12/2009. Most of those assessed lived in Bristol or Bath (64%, 258/405). Of the 405 patients, 61 (15%) were assessed in 2006, 96 (24%) in 2007, 132 (33%) in 2008, and 116 (29%) in 2009.

Of the 388 children for whom school attendance was recorded, 125 (32%) children were defined as having severe CFS/ME (housebound), 146 (38%) as having moderate CFS/ME and 117 (30%) as having mild CFS/ME.

Table 1 describes the characteristics of the 397 children whose mothers were not interviewed and the eight children whose mothers were interviewed. Children in the interviewed group were slightly younger than children in the non-interviewed group (11.9 vs 13.8 years, t test P = 0.03). The median (IQR) time-to-assessment was 9 (7.5 - 12.5) months for the interviewed group, and 13 (8 - 24) months for the non-interviewed group (Mann-Whitney P = 0.09). All other characteristics of children in the interviewed group were similar to those whose parents were not interviewed.

**Table 1:** Characteristics of children assessed by the RNHRD between 2006 and 2009 and characteristics of children interviewed for qualitative analysis.

	Interviewed		Not interviewed		P-value*
Female N (%)	8	5 (62.5%)	397	281 (70.8%)	0.70
Severity of illness N (%)					
Mild/moderate	8	4 (57.1%)	397	259 (68.0%)	0.72
Severe	8	3 (42.9%)	397	122 (32.0%)	
Age (years), mean (SD),	8	11.9 (4.3)	397	13.8 (2.5)	0.03
Fatigue (0 - 33), mean (SD)	8	24.3 (5.5)	382	24.0 (5.0)	0.90
SF-36 (0 - 100), mean (SD)	8	19.9 (5.9)	383	20.8 (5.0)	0.60
No. of Symptoms (0 - 14), mean (SD)	7	9.7 (2.1)	389	8.4 (2.5)	0.17
Depression (HADS) (0 - 21) mean (SD)	5	8.0 (4.6)	289	7.0 (3.6)	0.56
Anxiety (HADS) (0 - 21) mean (SD)	5	9.4 (6.5)	288	8.6 (4.2)	0.66
Anxiety (SCAS) (0 - 90) mean (SD)	7	32.7 (13.9)	358	29.7 (18.3)	0.66
Months to assessment, median (IQR)	8	9 (7.5 - 12.5)	397	13 (8 - 24)	0.09

* Fisher's exact test for comparison of proportions, Student's t test for comparison of mean values, Mann-Whitney two-sample test for comparison of medians.

### Number of children seen within the NICE recommended time frame

Of the 125 patients with severe CFS/ME only one was seen immediately (defined as less than three months after symptom onset); of those with mild or moderate CFS/ME, only 19% (49/263) were seen within 7 months of symptom onset. 97 children (24%) children had an extremely long delay (> 24 months) before assessment.

### Factors associated with time to assessment

Complete data on school attendance, fatigue, SF-36, HADS and SCAS were available for 246 of the 405 children (61%). Table 2 shows the hazard ratios from multivariable survival analysis showing the associations of time-to-assessment with patient characteristics. Fatigue was the only factor associated with shorter time-to-assessment (HR = 1.15; 95% CI 1.03, 1.29 per unit increase in Chalder fatigue score; P = 0.01). Age, gender, disability, anxiety (either HADS or SCAS), depression, total number of symptoms, and severity of illness were not associated with time-to-assessment.

**Table 2:** Hazard ratios from multivariable Cox regression model for the association of time-to-assessment with age, sex, fatigue, disability, anxiety, depression, and severity (school attendance) (N = 246).

	Hazard ratio (95% CI)	P-value
Age	0.96 (0.88, 1.04)	0.33
Sex (female vs male)	0.91 (0.68, 1.23)	0.55
Fatigue (Chalder) Score*	1.15 (1.03, 1.29)	0.01
Disability (SF-36)*	0.98 (0.87, 1.12)	0.79
Anxiety (HADS)*	0.97 (0.90, 1.05)	0.45
Depression (HADS)*	1.00 (0.92, 1.09)	0.94
Number of symptoms	0.94 (0.88, 1.01)	0.08
		
Severity of illness		0.62 (P_trend_)
Mild	1.00 (reference)	
Moderate	1.04 (0.74, 1.45)	0.82
Severe	1.11 (0.73, 1.67)	0.62

* These measures were re-scaled so that the range for each was approximately 0-10 to facilitate comparability in the multivariable model.

### Qualitative interviews

The parents of 11 children were invited to take part; of these three were new patients and eight were seen at follow-up. One was unable to attend due to weather conditions and a further two were unable to arrange an interview date due to practical problems. Of the nine parents who were interviewed, seven interviews were conducted with one parent present; one interview was conducted with a parent couple. All the parents were mothers apart from the parent couple where a mother and step-father attended.

### Parents' views on barriers for accessing healthcare and effect on family

The themes that emerged from the data included: 'Lack of Knowledge', 'Attitudes and Beliefs' and 'Communication Problems'. Three further themes described the effect of these barriers on the parent and family: 'Anger and Frustration', 'Conflict with the Medical Profession' and 'Delay in Diagnosis and Access to the Specialist Service'. All themes are described further below and further illustrated in Table 3.

**Table 3:** Parental quotes

Theme	Parents Quote
**Lack of Doctor Knowledge**	"I think the first thing is the diagnosis, but I think most people find that difficult. The Drs [GPs] aren't always aware..." Parent 8
	"She never heard of it in someone so young, we phoned social services - they'd never heard of it in someone so young. The doctor's [GPs] didn't hear of it, didn't know much about it, they said any leaflets we get could we pass on." Parent 3
	"I mean, he knew he needed to do something, he wasn't entirely sure what he needed to do...I gave him a little push!!" Parent 4 referring to the GP
	"He [GP] just said that she'd get tired and that sort of thing - everyone just thinks its tiredness when they don't sort of understand there's a lot of stuff that goes with it." Parent 3
	"But of course you know it takes ages to get a diagnosis, so it was sort of December before we got a diagnosis but they [Paediatrician] wouldn't refer her across to the ME unit here, and it just, you kind of need a lot of support to start with to know what you're doing and we just didn't get that." Parent 6
	"getting the wheelchair, the OTs, that sort of thing we struggled with...it was done through my knowledge... and I talk to a lot of people and a lot of people were giving me information. I had someone phone me to get a bit of help with the DLA, well we didn't even know we were entitled to it, no one said we could get a social worker who would deal with all this for us. Nobody told us anything." Parent 8

**Attitudes and Beliefs**	"Actually, he did not believe in ME and he had actually implied that in earlier visits and things...'he's putting it on, as far as I can see and as far as my team of people here believe he's strong...He's getting attention from being like this. He's obviously very depressed, very low" Parent 7 quoting Consultant Child Psychiatrist
	"It was just the attitude of the Drs [GP] and then it seemed like within the practice, once I'd asked doctors then they just spoke amongst themselves....... That frustrates me, I wish they would take a different approach to both mental illness and ME and everything." Parent 2
**Communication Doctors**	"nearly every month we were going [to the GP] and it was just, oh it's a virus she'll be alright - and she wasn't!" Parent 3
	"they [GP] talked down to you." Parent 2
	"just make you feel a little bit...they [GP] just make you feel inadequate sort of thing." Parent 3
	" The GPs and that they didn't really ask any questions and I wouldn't sort of, I didn't feel I could ask questions sort of thing." Parent 3
**Communication Parents**	"They [Child Psychiatry] wanted to know all about our home life, and they asked masses and masses of really probing questions and they would ask them in front of him, and I'm very honest with him but I just thought there were some questions that I wasn't going to answer in front of him. But they have this policy now that anything that's discussed is discussed in front of him so it felt like a lot of the things I was saying I had to censor 'cos I didn't want him to pick up. Also I was very aware that children can manipulate us you know so I didn't want to give him material to manipulate." Parent 7
	"I can't describe 100% what's going on inside my daughter, I can just relay... what I see and what she tells me" Parent 2
	"she said ' I can't see anything Adam' and she pulled her chair over and said 'why aren't you speaking? Come on Adam, tell me why aren't you speaking? Is something going on in school? Is something going on at home?' of course he didn't say anything. I'm right there and his dad's right there! And I thought, if something's going on at home...he's probably not going to say!" Parent 7
**Effect on Parent**	"So I did have a lot of sad, a lot of sadness because (the paediatrician) so wanted it to be (somatisation disorder) and I mean, I don't know if it is ME, if it is (somatisation disorder), but what's happening here is helping him, and that's all, I don't think the name, to me the name, I don't care about the name, I just want help for my child really. And to have to really fight, I don't think it's right really. And perhaps if my friends weren't so educated, I'd have been in a much worse situation really." Parent 7
	"my goodness, I'd come home [from the GP] in tears. I was just grateful my mum was with us at the time 'cos I'd just come home and be like you know, I'd doubt myself. 'Am I being an overbearing mum? Am I being too soft on her? I questioned all these. But at the same time I'd be so mad 'cos I'd look at her and see she wasn't my usual you know, bubbly girl." Parent 2
	".....so then I got a letter back which basically said that the *mother *was not being cooperative and all of this nonsense, so when I went to see the paediatrician. the next time he asked why aren't you being cooperative? If you want your son to get better you need to cooperate! So I said 'there's nothing to cooperate with!! You haven't offered me anything. This is just nonsense!'" Parent 7

#### 'Lack of Knowledge'

Parents felt both GPs and Paediatricians lacked knowledge of CFS/ME, were unsure how to make a diagnosis, and didn't understand the referral process or how to access practical support. They felt that GPs, in particular, knew little about the condition or the recommended guidelines when CFS/ME was suspected or diagnosed. This led to a delay in diagnosis and the parent having to inform the GP about the specialist service and referral criteria.

"It wasn't that they [General Paediatrician] didn't know about it...they didn't seem to have a real grasp or understanding. Not a real understanding." Parent 6

He [the GP] just said that she'd get tired and that sort of thing - everyone just thinks it's tiredness when they don't sort of understand there's a lot of stuff that goes with it." Parent 3

Parents felt they were dismissed by GPs as worrying over normal childhood illnesses and weren't signposted to the practical support they were entitled to.

"nearly every month we were going and it was just, oh it's a virus she'll be alright - and she wasn't!" Parent 3

One family said that friends had to tell them how to get a wheelchair and that there was a 'missing link' between the GP consultation and the specialist service, which led to a delay in receiving practical support.

"there's a connection missing somewhere and I don't know where it's got to be put in, but we need to be pushed in a direction." Parent 8

#### 'Attitudes and Beliefs'

Parents described problems with judgemental blaming attitudes by GPs, paediatricians and child psychiatrists. They described these attitudes as making them feel abandoned and disrespected. They specifically described doctors 'closing ranks', blaming their parenting, dismissing symptoms as fabrication and warning them of the stigma related to the diagnosis of CFS/ME. Parents felt these beliefs prevented the medical profession understanding the impact of the condition on the child and family.

"he's putting it on, as far as I can see and as far as my team of people here believe he's strong... he's getting attention from being like this ........... 'you don't want that on his medical records' that, that was a negative thing to have and that it just was going to be harmful."" Parent 7 quoting Consultant Child Psychiatrist

#### 'Communication Problems'

Within the theme of 'Communication Problems' two subthemes were identified; 'Doctor Communication Problems' and 'Parent Communication Problems'.

'Doctor Communication Problems':

Parents reported that GPs and in one case a Child Psychiatrist, delegitimized their child's experience, were patronising, didn't listen to them, and dismissed their concerns. They also failed to ask questions and empower their child to talk; nor did they express empathy. Parents reported having to attend the GP surgery on many occasions to convey the seriousness of the problem.

"you were sort of in then out at the doctors [GPs] - trying to fit as many in one day as they can sort of thing. Not listening."Parent 3

"I thought, no, you're not hearing what I'm saying. You're not, you're not listening really. So again I felt that it was dismissed but I was very grateful that there wasn't anything horrid going on obviously. But I did think that he could have possibly avoided having that and having her interrogate him if it had been a more recognisable symptom." Parent 7 referring to Child Psychiatrist

Parents felt that they were patronised and made to feel 'inadequate' as parents. They felt that lack of empathy was expressed both in the verbal communication with doctors and their facial expressions and body language.

"in the GP office, there was a scowl as you came in, 'Mrs X what can I do for you today?'" (Gruff, stern voice) Parent 2

Parents reported that they felt unable to ask questions and approach the GP because they felt dismissed.

"just make you feel a little bit...they just make you feel inadequate sort of thing...The GPs and that they didn't really ask any questions and I wouldn't sort of, I didn't feel I could ask questions sort of thing." Parent 3

Parents sometimes found their GPs' and paediatricians' attempts to give information (even if they knew something about the condition) were not always helpful, or were not given in a way they could understand it or put it into practice.

"they [General Paediatrician] sort of gave us, they told us about pacing. But it was very vague... it's very difficult, they talked about high level and low level activity but you know, in quite brief appointments it's very difficult to work out exactly what was meant..." Parent 6

'Parent Communication Problems':

Parents struggled to communicate an illness that wasn't visible as well as having difficulty communicating a problem that their child, and not themselves, were experiencing. They reported that their children found it hard to put their experiences into words and that it was difficult answering more probing questions in front of the child.

"I can't describe 100% what's going on inside my daughter, I can just relay... what I see and what she tells me, and it wasn't until later on when she was able to describe that I knew what it felt like. It wasn't 'til then I could understand what was going on at the time, she was unable to find the words." Parent 2

### Effect on Parents

Parents described 'anger and frustration' and 'conflict with the medical profession' as consequences of struggling to access health care for their child. 'Anger and frustration' occurred both in consultations and afterwards and was attributed to: their interactions with the medical profession; feeling helpless; their frustration of being unable to access care; not knowing where to turn to for support and practical advice and abandonment by the medical profession.

"My goodness, I'd come home in tears. I was just grateful my mum was with us at the time 'cos I'd just come home and be like you know, I'd doubt myself. 'Am I being an overbearing mum? Am I being too soft on her? I questioned all these. But at the same time I'd be so mad 'cos I'd look at her and see she wasn't my usual you know, bubbly girl." Parent 2

'Conflict with the medical profession' resulted from perceived delay in diagnosis and referral, disagreement over aetiology and blame of the child or the parents for the child's condition.

## Discussion

We have found that few children and young people with CFS/ME are seen within the time frames recommended by NICE [7]. Nearly one quarter of children waited over two years before assessment by a specialist service. Children who were housebound were no more likely to be seen earlier than those who attended school full-time despite NICE guidelines recommending urgent assessment. Other markers of severity such as physical disability, number of symptoms, co-morbid mood problems or pain did not affect time-to-assessment. These findings suggest that delays in diagnosis may be due to barriers in accessing services rather than patient need. This is consistent with the qualitative data where parents described problems with their own and clinician's lack of knowledge, the clinicians attitudes, and beliefs. All of this may have led to the considerable communication problems reported and appear to contribute to the barriers experienced. Barriers to accessing specialist services appear to increase frustration and distress for both the parents and the child with CFS/ME.

Some of the problems described could be due to their GP's lack of knowledge about CFS/ME. Bowen et al found that 48% of GPs were not confident in diagnosing CFS/ME in adults and 51% could not identify 3 key clinical features (fatigue, symptom exacerbation on over-exertion and marked fluctuation of symptoms) [17]. It seems likely that GPs would have similar uncertainty in the diagnosis and management in children. Some GPs feel that the label of CFS/ME can be potentially harmful for adult patients and they struggle to understand the role of secondary care, which may also lead to a delay in access to specialist services [18].

### Strengths and limitations

This is the first study to look at barriers for families trying to access specialist services for paediatric CFS/ME. Using quantitative and qualitative methods enabled us to investigate time-to-assessment using prospectively collected data on a large unselected group of children attending a paediatric CFS/ME clinic and to interview parents about the problems they experienced. The sample of children interviewed was representative of all children assessed by the service except in time-to-assessment (the non-interview group included children with very long delays before assessment) and age (the interview group included one 3-year-old child). As the sample interviewed was a convenience sample recruited consecutively from clinic, the differences are likely to be due to chance rather than bias. This study cohort was from a well-established regional specialist service with stable referral patterns. The problems described may not be generalisable to other areas where service provision is scarce. It might be expected that in an area with a less well-established service, the barriers to accessing healthcare would be different and the delay to accessing healthcare far longer. In addition, we do not have sufficient information from fathers who may experience and report different barriers to accessing care for their children. It would also be useful to observe the interactions between parents and clinicians at the referral stage in order to gain a greater understanding of the dynamics involved.

### Results in context of previous literature

This study is consistent with previous research in adults with CFS/ME who report problems such as a 'lack of acknowledgement', 'trivialisation of symptoms' and 'interpreting exhaustion as depression' [19,20]. These problems are acknowledged by health care professionals as impacting on recovery [21]. Teenagers also describe the results of delay in diagnosis as 'a difficult time' characterised by a lack of information, understanding and awareness of their condition [22].

Parents in this study described problems with judgemental blaming attitudes and beliefs which they felt prevented the medical profession understanding the impact of the condition, thus delaying referral to specialist services. This is consistent with studies in adults with CFS/ME who describe feeling blamed, dismissed and under pressure to 'convince' the medical profession of the reality of their illness thus delaying access to services [20,23,24]. Some GPs believe that the label of CFS/ME "could be harmful" [18] which may therefore delay diagnosis or referral. GPs themselves described their view of CFS/ME as "quite patronising" [18]. GPs also describe patients with CFS/ME as "transgressing the work ethic", "lacking stoicism" or "having certain personality trait" which they described "pejoratively" [25]. All of these factors may delay diagnosis and referral to specialist services.

Difficulties in parents' ability to communicate their child's symptoms is not unique for CFS/ME. Parents of children with complicated respiratory tract infections also experienced delay in accessing appropriate help because parents had difficulty communicating with doctors in both primary and secondary care [26]. Communication problems may be compounded by the lack of a biomarker or test that would help to explain the problem. This appears to contribute to confrontations between patients with CFS/ME, their parents and their doctors [24].

## Conclusions

Children will continue to experience delays in accessing specialist services unless GP's knowledge and attitudes towards CFS/ME changes. GPs and Paediatricians need to recognise the difficulties parents face, be aware of local care pathways and support families as they navigate the health care system. Further training for GPs and Paediatricians is needed to improve knowledge, maximise communication skills and expedite referral to specialist services.

## Competing interests

Dr Crawley is a Medical Advisor for the Association for Young people with ME.

## Authors' contributions

CW carried out the interviews and analysis of the qualitative data. TD, A HF and AS also analysed the qualitative data. SC performed the statistical analysis. EC conceived the study, participated in its design and co-ordination and helped draft the manuscript. All authors read and approved the final manuscript.

## Pre-publication history

The pre-publication history for this paper can be accessed here:

http://www.biomedcentral.com/1472-6963/11/308/prepub
