# Correction: What stops children with a chronic illness accessing health care: a mixed methods study in children with Chronic Fatigue Syndrome/Myalgic Encephalomyelitis (CFS/ME)

**DOI:** 10.1186/s12913-023-09485-6

**Published:** 2023-05-05

**Authors:** Carly M Webb, Simon M Collin, Toity Deave, Andrew Haig-Ferguson, Amy Spatz, Esther Crawley

**Affiliations:** 1 St George’s University of London, Cranmer Terrace, London, UK; 2 Centre for Child and Adolescent Health, School of Social & Community Medicine, University of Bristol, Bristol, UK; 3 Centre for Child and Adolescent Health, University of the West of England, Bristol, UK

**Correction to**: *BMC Health Services Research (2011) 11:308*. 10.1186/1472-6963-11-308.

Following a report of a publications review jointly commissioned by the Health Research Authority and the University of Bristol, the authors would like to correct the following element of the ethics statement provided in the original article [1]:

Ethical permission for the qualitative part of the study was granted by the North Somerset and South Bristol Research Ethics Committee (REC Reference number 09/H0106/81). The study was also approved by the Research and Development department of the RNHRD. The North Somerset & South Bristol Research Ethics Committee decided that the collection and analysis of these data for service evaluation did not require ethical review by an NHS research ethics committee or approval from the NHS R&D Office (REC reference number 07/Q2006/48).

The original article [1] has been corrected.
